# Precision Probiotics Regulate Blood Glucose, Cholesterol, Body Fat Percentage, and Weight Under Eight-Week High-Fat Diet

**DOI:** 10.3390/metabo15100642

**Published:** 2025-09-25

**Authors:** Jinhua Chi, Jeffrey S. Patterson, Lingjun Li, Nicole Lalime, Daniella Hawley, Kyle Joohyung Kim, Li Liu, Julia Yue Cui, Dorothy D. Sears, Paniz Jasbi, Haiwei Gu

**Affiliations:** 1 College of Health Solutions, Arizona State University, Phoenix, AZ 85004, USAjspatte5@asu.edu (J.S.P.); lingjun2@asu.edu (L.L.); dorothy.sears@asu.edu (D.D.S.); 2 School of Biological and Health Systems Engineering, Arizona State University, Tempe, AZ 85287, USA; nlalime@asu.edu; 3 School of Life Sciences, Arizona State University, Tempe, AZ 85287, USA; 4 Department of Environmental & Occupational Health Sciences, University of Washington, Seattle, WA 98195, USA; kk1109@uw.edu (K.J.K.);; 5 Systems Precision Engineering and Advanced Research (SPEAR), Theriome Inc., Phoenix, AZ 85004, USA; jasbi@therio.me; 6 MetaBiotics LLC, Scottsdale, AZ 85259, USA

**Keywords:** hyperglycemia, precision probiotics, blood glucose control, *Lactobacillus rhamnosus*, *Lactobacillus reuteri*, *Lactobacillus salivarius*

## Abstract

**Background/Objectives**: Poor glycemic control is reaching an epidemic prevalence globally. It is associated with significantly morbid health concerns including retinopathy, neuropathy, nephropathy, cancer, and cardiovascular disease. Probiotics have shown promise in reducing health complications associated with poor blood glucose control. We tested a novel approach to designing a precision probiotic cocktail for improving blood glucose homeostasis. **Methods**: We tested the in vitro glucose consumption rate of twelve mouse microbiome bacterial strains and selected three with the greatest glucose consumption for the probiotic cocktail. The in vivo metabolic impact of ingesting the selected probiotic cocktail was evaluated in twelve C57BL/6J male mice fed a high-fat diet for eight weeks. **Results**: Compared to a control group, the probiotic group (*L. rhamnosus*, *L. reuteri*, and *L. salivarius*) exhibited significantly lower blood glucose levels, body weight, and body fat percentage. Moreover, the probiotic cocktail also demonstrated the ability to reduce serum insulin, total cholesterol, very-low-density lipoprotein/low-density lipoprotein cholesterol, and total cholesterol to high-density lipoprotein ratio. For further mechanistic investigation, untargeted metabolomics analyses uncovered overall downregulations in energy substrates and producing pathways like gluconeogenesis, acylcarnitine synthesis, glycolysis, the mitochondrial electron transport chain, the TCA cycle, and the building blocks for ATP formation. Partial least squares-discriminant analyses also confirmed clear group differences in metabolic activity. 16S rRNA sequencing from extracted gut microbiota also showed significant increases in Faith’s phylogenetic diversity, *Lachnospiraceae bacterium 609-strain*, and the genus *Muribaculaceae* as well as group β-diversity differences after probiotic intake. **Conclusions**: As such, we successfully developed a blend of three probiotics to effectively reduce blood glucose levels in male mice, which could further mitigate adverse health effects in the host.

## 1. Introduction

Hyperglycemia, or high blood glucose levels, is reaching an epidemic proportion globally with significant and costly health concerns [1]. Nearly one-third of the United States population is estimated to suffer from poor glucose homeostasis, and this incidence is predicted to significantly increase over the next decade [1]. If left untreated, chronic hyperglycemia may result in life-threatening, chronic conditions such as retinopathy, neuropathy, nephropathy, cancer, cardiovascular disease (CVD), and diabetic ketoacidosis [2]. Hyperglycemia is also closely associated with obesity, metabolic syndrome, and a reduction in quality of life and lifespan [3,4]. Notably, individuals with obesity are susceptible to multiple severe, noncommunicable diseases like type 2 diabetes (T2D), CVD, and cancer [5,6]. In addition, hyperglycemia is present more frequently in individuals with T2D, and nearly 115 million Americans are estimated to have prediabetes or T2D [1].

Poor glycemic control has a multitude of factors, such as medication use, genetic conditions, illness, and stress, but it most commonly originates from poor daily lifestyle habits and excessive consumption of foods high in added sugar [5]. Increased daily sugar intake typically arises from poor dietary choices that are riddled with ultra-processed foods [7,8]. As a result, a high-sugar diet is often coupled with a high-fat diet, which can further contribute to impaired glycemic control by promoting insulin resistance and impairing glucose metabolism [9,10]. A recent study found that administering a high-fat and high-sugar diet just once per week for twelve weeks resulted in acquired insulin resistance and non-alcoholic fatty liver disease in C57BL/6J mice [11]. Efforts to combat this dietary trend and reduce hyperglycemic-related conditions are largely failing due to multifaceted complexities like food swamps and deserts [12,13]. As such, developing an alternative beyond the simple promotion of nutritional recommendations or expensive medications is becoming essential for blood glucose regulation.

At the molecular level, sugar is primarily broken down and circulated as glucose in the bloodstream for adenosine triphosphate (ATP) production in surrounding tissues [14,15]. When energy needs are met, glucose is then stored as glycogen for later use or converted to adipose tissue once glycogen stores are full [16,17]. The pancreatic hormone, insulin, is vital in regulating blood sugar due to its role in glucose cellular uptake [16,17]. Chronic consumption of added sugar can impair insulin binding and glucose transport into the cell, which may result in insulin resistance and worsened hyperglycemia over time [16,17]. In addition, excessive dietary fat, especially saturated and trans-fat, can lead to insulin resistance, impaired glucose metabolism, increased hepatic glucose production, and elevated blood sugar levels [9,10,18,19]. Notably, a poor diet has also been shown to have negative consequences in the gut. Recent evidence has shown that the high-fat and high-sugar Western diet increases intestinal permeability and elevates bloodstream endotoxin levels due to affected enterocyte tight junctions [20]. Similar poor dietary habits have also been associated with alterations in the gut microbiome composition, which is known to have important roles in metabolism, immunity, inflammation, and the gut–brain axis [21,22].

Probiotics, which have close interactions with the gut microbiome, are promising interventions to combat the health complications related to high blood sugar levels. A recent meta-analysis of seven studies involving adults with prediabetes found that probiotic supplementation significantly improved high-density lipoprotein cholesterol (HDL) and lowered the levels of glycated hemoglobin (HbA1c) and the homeostatic model assessment of insulin resistance (HOMA-IR). However, fasting blood glucose (FBG), low-density lipoprotein cholesterol (LDL), total cholesterol (TC), triglycerides (TG), and body mass index (BMI) were unchanged [23]. Several studies from another meta-analysis of probiotic supplementation among adults diagnosed with T2D did report significant improvements in FBG, HbA1c, and HOMA-IR [24]. This may be attributed to inconsistencies in bacterial strains or dosages and highlights the need for in vitro experimentation to objectively select strains and dosages to achieve the desired benefit. In addition, neither of the meta-analyses investigated the probiotic effects in metabolically healthy individuals. A review of studies aimed to develop probiotics for improved digestion, blood glucose, cholesterol, immunity, and inflammation determined that *Lactobacillus* and *Bifidobacterium* were largely the most effective in achieving the desired benefits [25]. Yet, the development of a precision probiotic using objectively selected bacterial strains and dosages to enhance gut glucose consumption for lowering of blood glucose levels in the host as an alternative therapy is needed in the current societal landscape. Furthermore, extensive screening of specific probiotics is necessary to determine the most effective strains or probiotic combinations to optimize performance.

In this study, we aim to develop a novel precision probiotic cocktail for increased gut glucose consumption that may improve blood glucose control and weight loss in the host. We hypothesize that bacterial strains with enhanced capabilities to consume glucose will effectively reduce the bioavailability of these sugars to the host. As a result, they may regulate blood glucose levels and potentially help mitigate adverse health effects of chronic high sugar intake. To fully investigate the metabolic effects of the precision probiotic cocktail, we will perform untargeted metabolomic analyses on collected serum and liver tissue using liquid chromatography-mass spectrometry (LC-MS) [26,27,28,29,30,31]. Additionally, mouse gut microbiota will be extracted for 16S rRNA sequencing to assess probiotic interactions and microbiome health [32]. Our objective is to demonstrate how probiotic intake can modulate the negative effects of a high-fat diet in elevating blood glucose levels and potentially improve host weight maintenance and biomarkers of metabolic health. In doing so, the risk for developing hyperglycemic-related chronic conditions can be potentially attenuated.

## 2. Materials and Methods

### 2.1. Bacterial Culture of Probiotic Strains

The following bacterial strains were purchased from American Type Culture Collection (ATCC, Manassas, VA, USA): *Lactobacillus acidophilus* (*L. acidophilus*; ATCC NO. 4356), *Lactobacillus casei* (*L. casei*; ATCC NO. 393), *Lactobacillus gasseri * (*L. gasseri*; ATCC NO. 33323), *Lactobacillus plantarum * (*L. plantarum*; ATCC NO. 14917), *Lactobacillus paracasei * (*L. paracasei*; ATCC NO. 334), *Lactobacillus rhamnosus * (*L. rhamnosus*; ATCC NO. 7469), *Lactobacillus reuteri * (*L. reuteri*; ATCC NO. 23272), *Lactobacillus salivarius * (*L. salivarius*; ATCC NO. 11741), *Bifidobacterium animalis * (*B. animalis*; ATCC NO. 27536), *Bifidobacterium bifidum * (*B. bifidum*; ATCC NO. 29521), and *Bifidobacterium longum * (*B. longum*; ATCC NO. 15707). Nissle 1917 was generously provided by Dr. Xuan Wang’s lab in the School of Life Sciences at Arizona State University (Tempe, AZ, USA).

Bacteria were cultured at 37 °C in an anaerobic chamber (Whitley Workstation DG250, Microbiology International) filled with 80% N_2_, 10% H_2_, and 10% CO_2_. de Man Rogosa and Sharpe (MRS) Broth (MilliporeSigma, Burlington, MA, USA) and Gifu Anaerobic Broth (GAM, HiMedia, Kennett Square, PA, USA) are commonly used culture media for the bacteria included in this study, and they were equilibrated in the anaerobic environment prior to use. After 24 h of incubation at 37 °C, the whole bacterial sample was centrifuged at 5000× *g* for 5 min, and the supernatant was collected for further measurement. Approximately 20 μL of medium was used to measure glucose levels with a glucometer (OneTouch Ultra 2 Blood Glucose Meter Kit, LifeScan, Milpitas, CA, USA).

### 2.2. Animals and Probiotic Cocktail

To prepare the probiotic cocktail, *L. rhamnosus*, *L. reuteri*, and *L. salivarius* were cultured in GAM Broth under 37 °C in an anaerobic chamber (Whitley Workstation DG250, Microbiology International) filled with 80% N_2_, 10% H_2_, and 10% CO_2_. After 24 h of incubation at 37 °C, bacteria were collected and freeze-dried at −80 °C. Before oral gavage was performed on the mice, freeze-dried probiotics were thawed and diluted with sterile PBS to 5 × 10^8^ CFU/100 μL.

All mice were housed according to the Association of Assessment and Accreditation of Laboratory Animal Care International guidelines, and our studies were approved by the Institutional Animal Care and Use Committee at Arizona State University (Protocol Number: 24-2071R, Approved on 28 March 2024). Seven-week-old C57BL/6J male mice were purchased from Charles River Labs (Wilmington, MA, USA) and acclimatized for a week. The mice were then randomized to either the probiotic or control group. They were housed under 23 °C on a 12 h light/dark cycle in standard open cages. The mice had free access to a 45% high-fat diet (D12451, Research Diets, New Brunswick, NJ, Canada) and water for 8 weeks. Each mouse was randomly assigned to either the control group or the probiotic treatment group by blinded researchers. A total of five mice were housed in standardized cages that were identical in cage density, structure, and position. After 1 week of adaptation, each group received either bacterial suspensions or the vehicle (sterile PBS) three times per week (every other day). Bacterial cells of *L. rhamnosus*, *L. reuteri*, and *L. salivarius* were administered via oral gavage in a 100 μL (5 × 10^8^ CFU/100 μL each) suspension. The vehicle, sterile PBS, was administered to the control group using the same procedure. At the end of the 8-week study, the mice were euthanized after a 16 h fasting period, and blood and tissues were harvested for analysis. Serum was isolated from fasting whole blood and stored at −80 °C before assays were run. Investigators performing measurements were blinded to group allocation.

### 2.3. Glucose and Biomarker Measurement

Blood glucose measurements were conducted consistently after the last administration of the bacterial cocktail each week. After fasting for 6 h, the tips of the mouse tails were nicked with sharp scissors to collect blood by direct flow or by gently squeezing the tail. The glucometer (OneTouch Ultra 2 Blood Glucose Meter Kit, LifeScan, Milpitas, CA, USA) was placed against the tail tip, and the blood glucose levels were measured. Serum high-density lipoprotein (HDL) and very-low-density lipoprotein/low-density lipoprotein (VLDL/LDL) were measured using the HDL and VLDL/LDL Assay Kits (Sigma-Aldrich, St. Louis, MO, USA). Serum total cholesterol (TC) and triglyceride (TG) levels were measured with the LabAssay™ Cholesterol and Triglyceride Assay Kits (Wako Chemicals USA, Richmond, VA, USA). Serum insulin was measured with an Ultra-Sensitive Mouse Insulin ELISA Kit (Crystal Chemical, Ivyland, PA, USA).

### 2.4. Body Composition (EchoMRI)

Changes in body composition were assessed using magnetic resonance imaging (EchoMRI, Houston, TX, USA). MRI measurements were performed by placing each mouse into a thin-walled glass cylinder with an additional cylindrical insert to limit movement of the mouse. Mice were briefly submitted to a low-intensity electromagnetic field. Fat mass, lean mass, free water, and total water were measured.

### 2.5. Untargeted Metabolomics

Acetonitrile (ACN), methanol (MeOH), formic acid, and water, all LC-MS grade, were purchased from Fisher Scientific (Pittsburgh, PA, USA). DI water was provided in-house by a Water Purification System from EMD Millipore (Billerica, MA, USA). PBS was purchased from Pierce Biotechnology (Waltham, MA, USA). The internal standards (^13^C_3_-lactate and ^13^C_5_-^15^N-glutamic acid) were purchased from Cambridge Isotope Laboratories (Tewksbury, MA, USA).

#### 2.5.1. Serum Sample Preparation

Frozen serum samples were thawed overnight at 4 °C before 50 μL of each sample was placed into a 1.5 mL Eppendorf (EP) tube. The initial step for protein precipitation and metabolite extraction was performed by adding 500 μL MeOH and 50 μL internal standard solution (containing ^13^C_3_-lactate and ^13^C_5_-^15^N-glutamic acid). The mixture was then vortexed for 10 s and stored at −20 °C for 20 min, followed by centrifugation at 14,000 RPM for 10 min at 4 °C. The supernatants (450 μL each) were collected into new EP tubes and dried using a Vacuum Centrifuge drier (Eppendorf, Hamburg, Germany). The dried samples were reconstituted in 200 μL of H_2_O:PBS:ACN (2:2:6). The quality control (QC) was a pooled mixture of all serum samples.

#### 2.5.2. Liver Sample Preparation

Frozen liver tissue samples (~20 mg each) were thawed and homogenized in 200 µL MeOH:PBS (4:1, *v*:*v*, containing 1810.5 μM ^13^C_3_-lactate and 142 μM ^13^C_5_-^15^N-glutamic acid) in an EP tube using the Qagen TissueLyser II (Hilden, Germany). Then, 800 µL MeOH:PBS (4:1, *v*:*v*, containing 1810.5 μM ^13^C_3_-lactate and 142 μM ^13^C_5_-^15^N-glutamic acid) was added. After vortexing for 10 s, the samples were stored at −20 °C for 30 min. The samples were then sonicated in an ice bath for 10 min. The samples were centrifuged at 14,000 RPM for 15 min (4 °C), and 800 µL supernatant was transferred to a new EP tube. The samples were then dried under vacuum using a Vacuum Centrifuge drier (Eppendorf, Hamburg, Germany). Prior to MS analysis, the obtained residue was reconstituted in 300 μL H_2_O:PBS:ACN (2:2:6) and then filtered using 0.2 µm membrane filters. The QC sample was pooled from all the study samples.

#### 2.5.3. LC-MS

The untargeted LC-MS metabolomics method used in this study was modeled after a developed approach that has been used in a growing number of studies [32,33,34,35]. A Thermo Fisher Vanquish UPLC system coupled with an Orbitrap Explorise 240 mass analyzer was utilized to perform untargeted metabolomics analysis. A pooled QC sample was injected every 10 samples to ensure instrument consistency with an injection volume of 1 µL for both positive and negative ionization modes. Chromatographic separation was achieved using a Waters XBridge BEH Amide column (2.1 × 50 mm, 2.5 µm, Waters Corporation, Milford, MA, USA) maintained at 40 °C. The mobile phase consisted of Solvent A (0.1% formic acid in water) and Solvent B (0.1% formic acid in ACN), with a flow rate of 0.3 mL/min. Gradient elution was performed as follows: 0–0.5 min, 90% of B; 0.5–6.5 min, 90–40% of B; 6.5–9.0 min, 40% of B; 9.0–9.5 min, 40–90% of B; 9.5–15 min, 90% of B. The Orbitrap resolution was set to 120,000 with a full scan range (*m*/*z*) of 70–800. Full scan ddMS2 and AcquireX data acquisition with the Orbitrap resolution set at 60,000 were employed to acquire the MS/MS fragmentation pattern of detected precursor ions for metabolite identification.

To identify peaks in the MS spectra, we made extensive use of the in-house chemical standards (~600 aqueous metabolites) and additionally searched the spectra against the HMDB library, Lipidmap database, METLIN database, and commercial databases including mzCloud, Metabolika, and ChemSpider. The absolute intensity threshold for the MS data extraction was 1000, and the mass accuracy limit was set to 5 ppm. Identifications and annotations used available data for retention time (RT), exact mass (MS), MS/MS fragmentation pattern, and isotopic pattern. We used the Thermo Compound Discoverer 3.3 software for aqueous metabolomics data processing. To improve rigor, only the signals/peaks with a coefficient of variation (CV) < 20% across QC pools and the signals showing up in >80% of all the samples were included for further analysis. As a result, a total of 586 metabolites were analyzed for the serum samples, while 875 compounds were assessed in the liver tissue samples.

### 2.6. 16S rRNA Gene Sequencing Analysis

Fecal samples were collected from 16-week-old C57BL/6J male mice at the time of sacrifice. Genomic bacterial DNA was extracted and purified from the fecal samples using the E.Z.N.A.^®^ Stool DNA Kit (Omega Bio-Tek, Norcross, GA, USA) according to the provided instructions. DNA concentrations were determined using the Multimode Microplate Reader (BioTek, Winooski, VT, USA).

DNA extractions were sent to Novogene (Sacramento, CA, USA) for Illumina MiSeq amplicon sequencing of the bacterial 16S rRNA gene using slightly modified primers targeting the V3–V4 region for PCR amplification: 341F (5′-barcode-CCTAYGGGRBGCASCAG-3′) and 805R (5′-barcode-GGACTACNNGGGTATCTAAT-3′). Bacterial 16S rRNA amplification and sequencing were performed using a HiSeq-2500 sequencing system (250 bp paired-end; *n* = 4 per group). DADA2 [36] was used to collectively analyze sequences in Quantitative Insights Into Microbial Ecology (QIIME2) [37]. Multiple Python scripts (version 3.13) were developed to analyze raw data in FASTQ format in QIIME2. These scripts performed tasks such as measuring α-diversity and β-diversity, selecting operational taxonomic units (OTU), assigning sample sequences, filtering low-quality reads, converting formats, and generating visualizations.

### 2.7. Statistical Analysis

We obtained an estimate of the population standard deviation of the variable (s) and the magnitude of difference (d) that we expect to detect, and the sample size is given by *n* = 1 + 2C (s/d)^2^. C is a constant dependent on α and β, and equals 10.51 when α = 0.05, and 1 − β = 0.90. For a potential marker, we expect that s/d = 0.44, resulting in a needed sample size for both control and treatment of *n* = 1 + 2 × 10.51 × (3/6.5)^2^ = 5.48 (*n* = 6 mice per group per sex). Independent samples *t*-tests were performed to examine between group differences, respectively, for bacterial glucose consumption, host blood glucose, body weight, body fat percentage, insulin, HDL, VLDL/LDL, total cholesterol, and triglyceride levels. Paired samples *t*-tests were performed to examine within group differences for fasting blood glucose and body weight in the control and probiotic groups at baseline and eight weeks. Univariate and multivariate statistical analyses, including partial least squares-discriminant analysis (PLS-DA), volcano plots, and pathway and enzyme enrichment analyses were performed using the MetaboAnalyst 6.0 software [38]. QIIME2 was used to calculate the α-diversity using Faith’s phylogenetic diversity (the branch-length sum of the sample’s phylogeny). A threshold α-level of 0.05 was used to define statistical significance and metabolites were included based on *p*-value significance.

## 3. Results

### 3.1. In Vitro Glucose Consumption by Probiotic Strains

**Figure 1:**
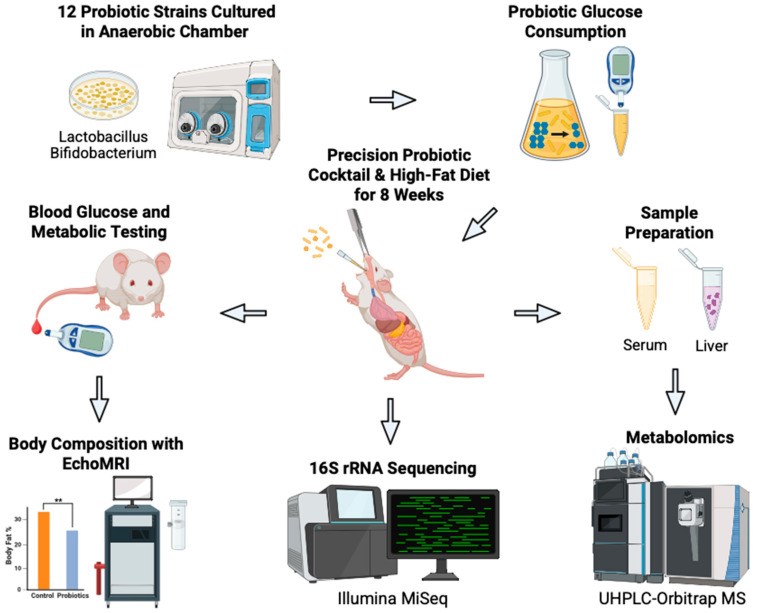
Schematic overview of our novel approach to develop precision probiotics for blood glucose control. Bacterial strains (*n* = 12) were tested in vitro to identify strains with the greatest glucose consumption capabilities. The top three strains were combined to produce the probiotic cocktail. The efficacy of the cocktail was then tested in vivo on C57BL/6J male mice on a high-fat diet who received oral gavages of the probiotics (*n* = 6) or vehicle PBS (*n* = 6) every other day for eight weeks. Body weight and blood glucose concentration were measured weekly. Terminal body composition was measured at eight weeks. Terminal serum and liver samples were harvested for metabolomic analysis. Fecal samples were collected for 16S rRNA sequencing and analysis. Created in BioRender. Patterson, J. (2025). Accessed on 3 July 2025. https://BioRender.com/bjkw2oe.

**Figure 2:**
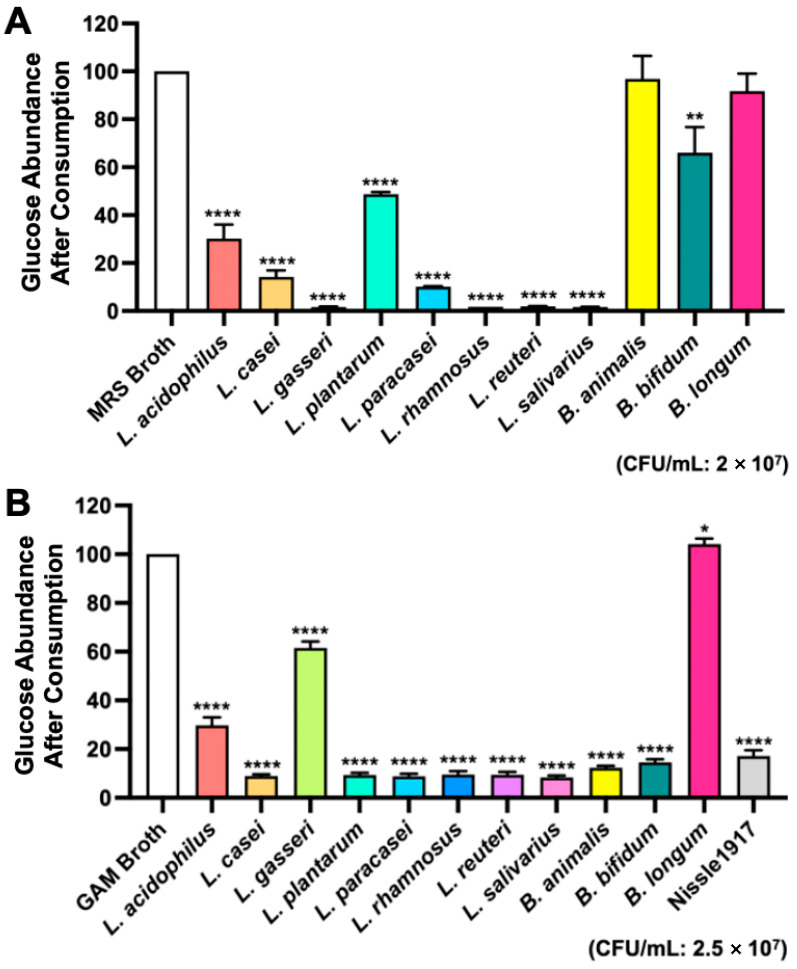
24 h glucose consumption by bacterial strains in vitro. Probiotics were incubated in (**A**) MRS Broth at a pH of 6.0 with a CFU/mL of 2 × 10^7^, or (**B**) GAM Broth at a pH of 6.99 with a CFU/mL of 2.5 × 10^7^. The graphs are presented as mean ± std. **** *p* < 0.0001, ** *p* < 0.01, * *p* < 0.05 vs. sterile broth, respectively.

Our objective was to develop a novel precision probiotic cocktail that would increase gut microbial glucose consumption and improve blood glucose regulation. Figure 1 shows a schematic overview of the study. Twelve mouse gut microbiota bacterial strains were obtained and tested for glucose consumption in vitro. MRS and GAM broths are commonly used culture media for the bacteria included in this study. Nissle, 1917 was only measured in the GAM broth due to it rarely growing in MRS. Glucose consumption by each bacterial strain was measured using a glucometer after 24 h incubation in MRS and GAM broths (Figure 2). In the MRS broth, glucose depletion was significantly greater in the samples inoculated with *L. acidophilus*, *L. casei*, *L. gasseri*, *L. plantarum*, *L. paracasei*, *L. rhamnosus*, *L. reuteri*, and *L. salivarius* (*p* < 0.0001) and *B. bifidum* (*p* < 0.01) compared to the uninoculated blank (Figure 2A). The samples containing *L. gasseri*, *L. rhamnosus*, *L. reuteri*, and *L. salivarius* exhibited the greatest glucose consumption with nearly all available glucose consumed. In the GAM broth, the depletion of glucose was significantly greater in the samples inoculated with *L. acidophilus*, *L. casei*, *L. gasseri*, *L. plantarum*, *L. paracasei*, *L. rhamnosus*, *L. reuteri*, *L. salivarius*, *B. animalis*, *B. bifidum*, and Nissle 1917 (*p* < 0.0001), while the glucose consumption of *B. longum* was also significant (*p* < 0.05) (Figure 2B). Interestingly, *L. gasseri* had the second highest remaining glucose abundance, but *L. rhamnosus*, *L. reuteri*, and *L. salivarius* had once again the highest glucose depletion.

### 3.2. In Vivo Testing of Precision Probiotics in C57BL/6J Mice

**Figure 3:**
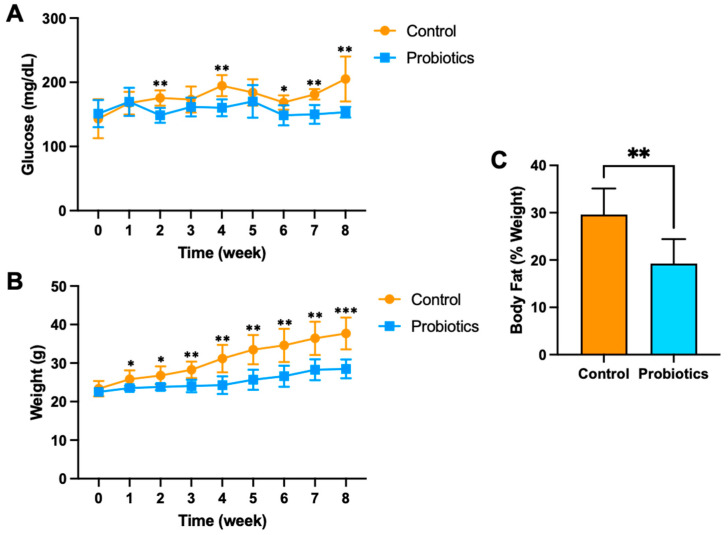
(**A**) Fasting blood glucose concentration, (**B**) body weight, and (**C**) body fat percentage of C57BL/6J male mice on a high-fat diet. Mice received the precision probiotics (*n* = 6; blue) or vehicle PBS (*n* = 6; orange) via oral gavage for eight weeks. The graphs are presented as mean ± std. *** *p* < 0.001, ** *p* < 0.01, * *p* < 0.05.

Based on the in vitro results, three high glucose-consuming bacterial strains were selected for in vivo testing in the precision probiotic cocktail in C57BL/6J male mice fed a high-fat diet over eight weeks (*n* = 6 per group). The mouse high-fat diet models the common U.S. dietary pattern and is known to increase body weight and impair blood glucose regulation [9,10,18,19]. Mice were gavaged with 100 μL of saline or the probiotic cocktail every other day during the eight weeks. The probiotic cocktail included *L. rhamnosus*, *L. reuteri*, and *L. salivarius* (5 × 10^8^ CFU/100 μL). Beyond week 1 of high fat feeding, the probiotic group demonstrated consistent and lower fasting blood glucose compared to the control group (Figure 3). Mean fasting blood glucose concentrations were significantly lower in the probiotic group compared to the control group at week 2 (148.5 ± 13.1 and 175.5 ± 11.9 mg/dL, respectively), week 4 (160.3 ± 13.1 and 194.7 ± 16.4 mg/dL), week 6 (148.5 ± 15.6 and 168.5 ± 11.1 mg/dL), week 7 (150.0 ± 14.5 and 181.2 ± 8.1 mg/dL), and week 8 (153.3 ± 8.2 and 205.2 ± 35.0 mg/dL) (Figure 3A). In addition, fasting blood glucose at week 8 was significantly increased compared to baseline in the control group (*p* = 0.008), while the probiotic group was relatively unchanged (*p* = 0.79).

Body weight was also relatively consistent and significantly lower in the probiotic group at weeks 1–8 when compared to the control group (Figure 3B). The magnitude of weight gain differences between the groups grew over weeks 3–8, suggesting continued beneficial effects and potential long-term benefits. Mean body weight was significantly lower in the probiotic group compared to the control group at week 2 (23.8 ± 0.8 and 26.8 ± 2.4 g, respectively), week 3 (24.1 ± 1.6 and 28.3 ± 2.1 g), week 4 (24.3 ± 2.3 and 31.2 ± 3.6 g), week 5 (25.7 ± 2.6 and 33.5 ± 3.8 g), week 6 (26.6 ± 2.7 and 34.6 ± 4.3 g), week 7 (28.3 ± 2.7 and 36.4 ± 4.3 g), and week 8 (28.5 ± 2.4 and 37.7 ± 4.1 g). Both groups demonstrated significant increases in body weight after being fed a high-fat diet for eight weeks, but the control group resulted in the most significant gain from baseline (*p* < 0.001). Terminal adiposity in the probiotic group was nearly 33% lower than in the control group when measured after eight weeks (*p* < 0.001) (Figure 3C).

**Figure 4:**
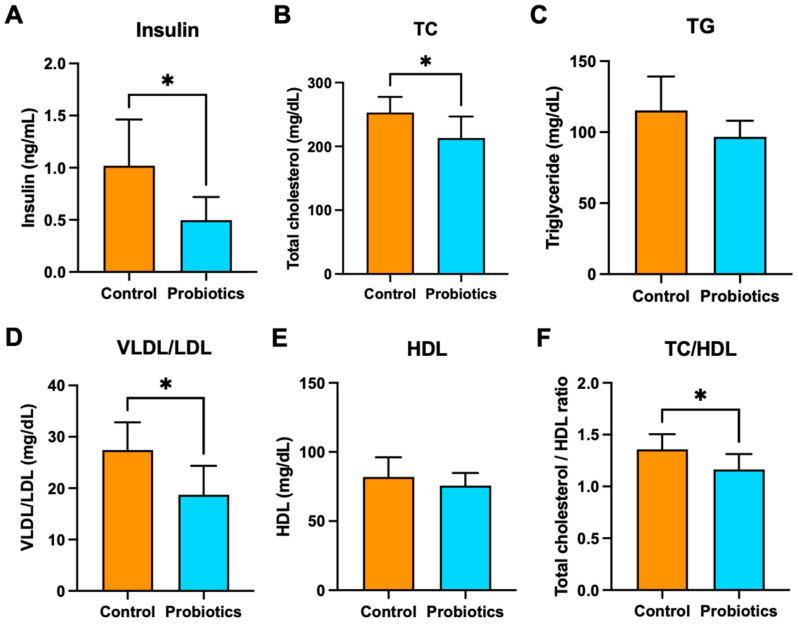
Terminal, fasting serum (**A**) insulin, (**B**) TC, (**C**) TG, (**D**) VLDL/LDL, (**E**) HDL, and (**F**) TC/HDL. The graphs are presented as mean ± std. * *p* < 0.05.

Effects of the probiotic cocktail on additional circulating biomarkers of metabolic health were measured. Terminal fasting serum insulin, total cholesterol (TC), triglyceride (TG), very-low-density and low-density lipoprotein (VLDL/LDL), and high-density lipoprotein (HDL) levels in the groups are shown in Figure 4. Insulin levels were nearly 50% lower (*p* < 0.05) in the probiotic group compared to the controls (Figure 4A). The probiotics also appeared to have a beneficial effect on TC, TG, and VLDL/LDL concentrations, which were all significantly lower compared to the controls (*p* < 0.05) (Figure 4B–D). There was no significant difference in HDL concentrations between the two groups (Figure 4E), but the probiotic group had a significantly lower TC/HDL ratio (*p* < 0.05) (Figure 4F).

### 3.3. Serum Untargeted Metabolomics

**Figure 5:**
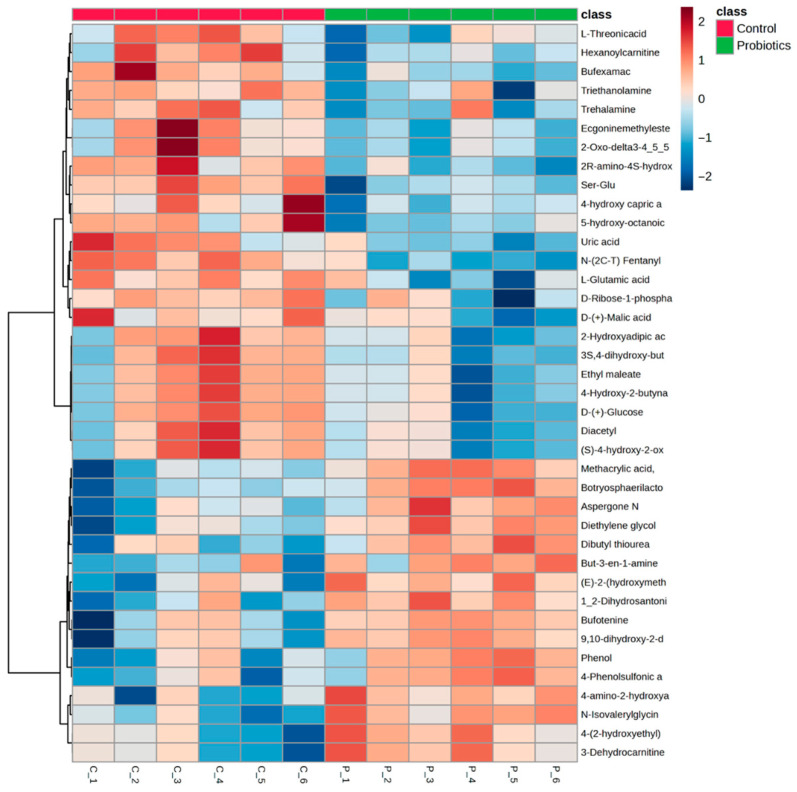
Heatmap of the significant metabolites (*p* < 0.05) from the terminal serum samples comparing the control and probiotic groups determined by independent samples *t*-tests.

For mechanistic investigation, terminal fasting serum samples from the C57BL/6J mice were used for untargeted metabolomics analysis. Sixty-two metabolites were significantly different in concentration between the two groups (Table S1, *p* < 0.05). Of these, carnitine derivatives essential for long chain fatty acid β-oxidation (3-dehydrocarnitine, hexanoylcarnitine, 2-ethylacryloylcarnitine, and O-propanoylcarnitine) and energy producing substrates (D-glucose, pyruvic acid, and D-mannose) were identified. Metabolites related to ATP formation (D-ribose-1-phosphate) and products of vitamin C metabolism (L-threonic acid) were also identified as significantly different between groups, along with several glycine and glutamate derivatives. Metabolite alterations and directionality can be observed in the Figure 5 heatmap. A volcano plot of control and probiotic serum metabolite analysis (fold change: probiotic/control) is shown in Figure S1.

**Figure 6:**
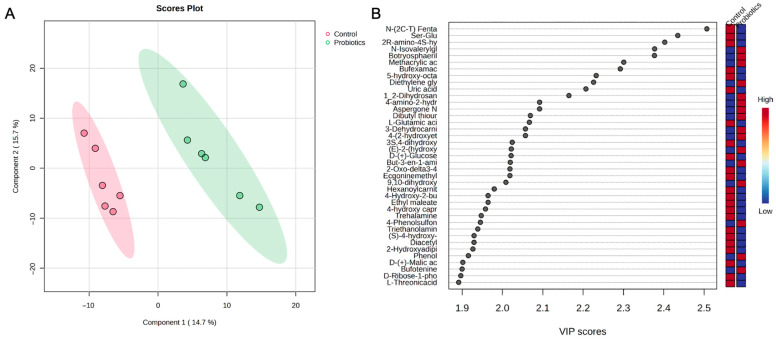
(**A**) PLS-DA score plot and (**B**) variable importance projection scores (VIP) comparing serum samples from the control and probiotic groups. Each dot in the PLS-DA score plot represents a serum sample at 8 weeks from each group. The directionality and influence of metabolites are depicted as a VIP score.

A partial least squares-discriminant analysis (PLS-DA) of the groups was also performed to assess group differences (Figure 6). In Figure 6A, the score plot demonstrates a clear separation between the groups. The top metabolites from the variable importance projection (VIP) scores are shown in Figure 6B. Metabolites such as N-isovalerylglycine, botryosphaerilactone A, and diethylene glycol diglycidyl ether are important for the probiotic grouping, while the medium chain fatty acid, 5-hydroxy-octanoic acid, and derivatives of amino acids like dipeptide ser-glu and 2R-amino-4S-hydroxy-5-hexynoic acid moved the controls in an opposing direction.

**Figure 7:**
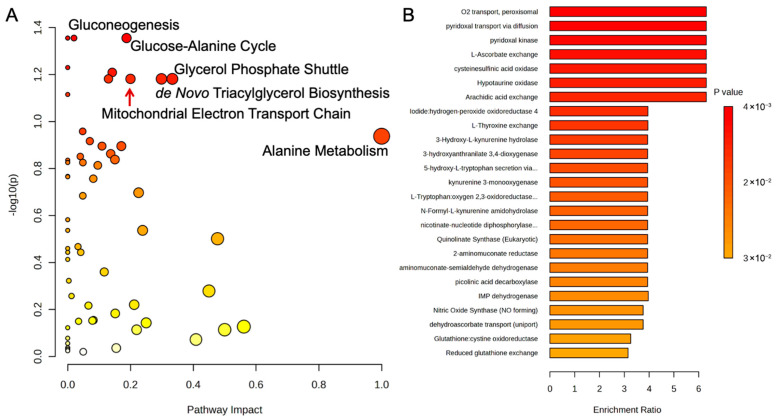
(**A**) Metabolic pathway analysis and (**B**) enrichment analysis of estimated enzyme activity of serum metabolites using MetaboAnalyst 6.0. The metabolic pathways are represented as circles according to their scores of enrichment (vertical axis, shade of red) and topology (pathway impact, horizontal axis, circle diameter). Enzyme enrichment is plotted as enrichment ratio, and more significant *p*-values are denoted by a darker shade of red.

A serum metabolic pathway analysis of the control and probiotic groups after 8 weeks was conducted and uncovered several pathways related to energy production (Figure 7A). The increases in gluconeogenesis (*p* = 0.04, Impact = 0.05), which is the endogenous production of glucose, and the glucose-alanine cycle (*p* = 0.04, Impact = 0.20) were closely associated with pyruvic acid, an important glycolysis substrate. In addition, the mitochondrial electron transport chain, the final step in oxidative phosphorylation and ATP production, was significantly affected (*p* < 0.05, Impact = 0.21). Fat metabolic pathways like the glycerol phosphate shuttle (*p* < 0.05, Impact = 0.33) and de novo triacylglycerol biosynthesis (*p* < 0.05, Impact = 0.30) were significantly associated with changes in glycerol-3-phosphate. An enrichment analysis of estimated enzyme activity was performed to further investigate the metabolic effects of the probiotic strains (Figure 7B). Oxygen transport within the peroxisomes (*p* < 0.05, Enrich > 6.0), organelles tasked with many functions related to β-oxidation and oxidative stress, was the most impacted, while arachidic acid exchange, a long-chain fatty acid, was also affected (*p* < 0.05, Enrich > 6.0). Additionally, enzymes related to pyridoxine (vitamin B6) and L-ascorbate exchange (vitamin C) were also altered (both *p* < 0.05, Enrich > 6.0), potentially due to reductions in the vitamin C metabolite, L-threonic acid, in the probiotic group.

### 3.4. Liver Untargeted LC-MS Metabolomics

**Figure 8:**
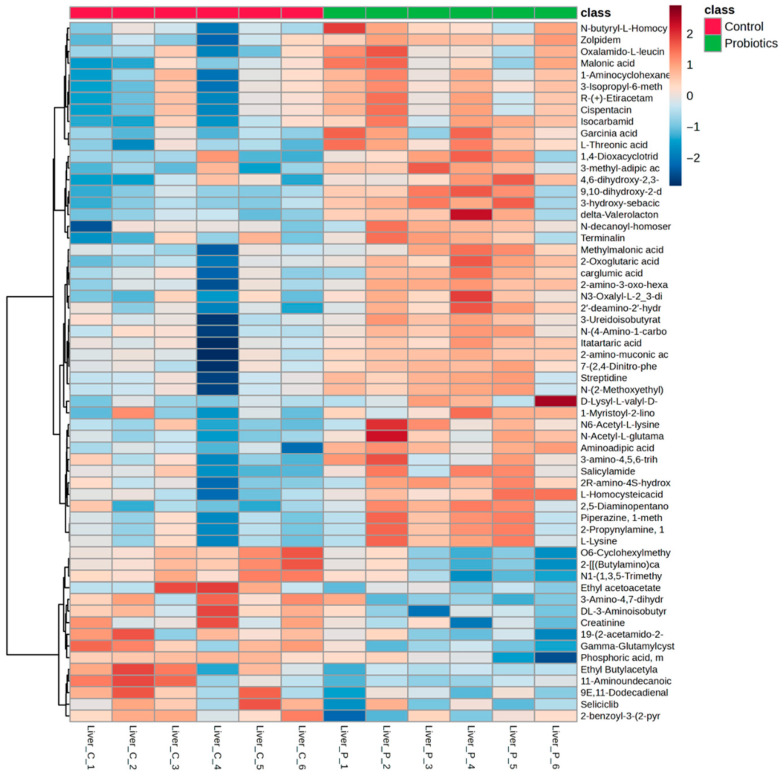
Heatmap of the significant liver metabolites (*p* < 0.05) comparing the control and probiotic groups.

The liver is essential in numerous bodily processes related to digestion, immune health, and detoxification [39]. It is also continuously tasked with maintaining blood glucose homeostasis, particularly when glucose intake is low [40,41]. Liver samples from the C57BL/6J mice were collected to further examine tissue metabolite effects of the precision probiotic cocktail. Fifty-nine metabolites were identified as significantly different between the two groups (Table S2, *p* < 0.05). Metabolite differences and directionality can be observed in Figure 8. The vitamin C derivative, L-threonic acid, had a higher liver abundance in the probiotic group compared to the control group, in contrast to the lower than control group level of this metabolite observed in the probiotics group serum. Oxoglutaric acid, a tricarboxylic acid (TCA) cycle intermediate, was also higher in the probiotic group compared to the control group. Compared to the control group, significant alterations to amino acid metabolism were observed in the probiotic group, including higher levels of L-lysine, N6-Acetyl-L-lysine, methylmalonic acid, and aminoadipic acid, but lower levels of the waste product creatinine. Several fatty acids were also different in the probiotic group compared to the control group, including 3-methyl-adipic acid, 9,10-dihydroxy-2-decenoic acid, and the fatty acid containing phospholipid, 1-myristoyl-2-linoleoyl-sn-glycero-3-phosphocholine. A volcano plot of the control and probiotic liver tissues (fold change: probiotic/control) is shown in Figure S2.

**Figure 9:**
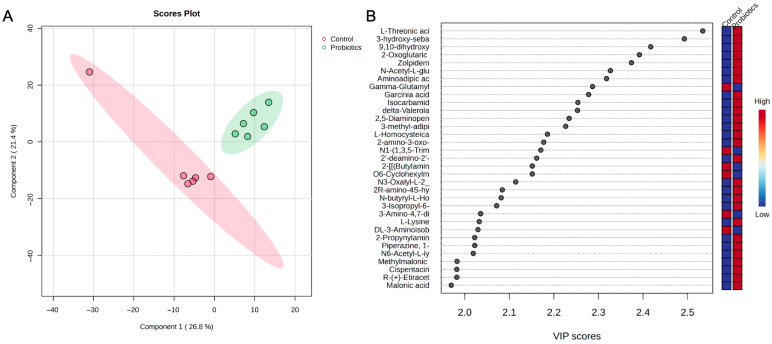
(**A**) PLS-DA score plot and (**B**) variable importance projection scores (VIP) comparing liver tissue samples from the control and probiotic groups. Each dot in the PLS-DA score plot represents a liver tissue sample at 8 weeks from each group. The directionality and influence of metabolites are depicted as a VIP score.

A PLS-DA of the control and probiotic liver samples was also conducted, and as shown in Figure 9A, the score plot depicts distinct group separation and tight clustering of the probiotic group. The top metabolites driving the separation from the VIP scores are depicted in Figure 9B. Similarly to the heatmap findings, L-threonic acid, oxoglutaric acid, N-acetyl-L-glutamate, aminoadipic acid, 3-methyl-adipic acid, L-lysine, and malonic acid were some of the most influential metabolites in the score plot.

**Figure 10:**
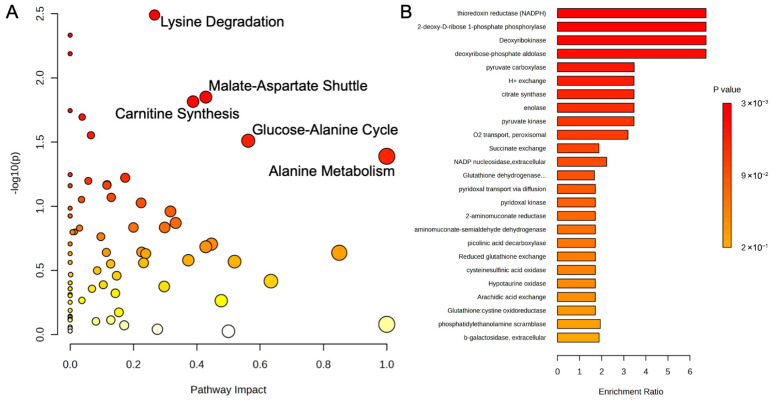
(**A**) Metabolic pathway analysis and (**B**) enrichment analysis of estimated enzyme activity of liver metabolites using MetaboAnalyst 6.0. The metabolic pathways are represented as circles according to their scores of enrichment (vertical axis, shade of red) and topology (pathway impact, horizontal axis, circle diameter). Enzyme enrichment is plotted as enrichment ratio, and more significant *p*-values are denoted by a darker shade of red.

A metabolic pathway analysis of liver tissue from the control and probiotic groups was performed and uncovered several pathways related to energy production and amino acid metabolism (Figure 10A). Metabolites like L-lysine, oxoglutaric acid, succinic acid, S-adenosylmethionine, and S-adenosylhomocysteine were implicated in the alterations of carnitine synthesis (*p* = 0.02, Impact = 0.43), while pyruvic acid and oxoglutaric acid were associated with the glucose-alanine cycle (*p* = 0.03, Impact = 0.56). Lysine degradation (*p* = 0.003, Impact = 0.27) substrates and intermediates like L-lysine, saccharopine, and aminoadipic acid were identified, while L-aspartic acid and glyoxylic acid were significant in the malate-aspartate shuttle (*p* = 0.01, Impact = 0.43) and alanine metabolism (*p* = 0.03, Impact = 1.0), respectively. An enrichment analysis of estimated enzyme activity was also conducted in the liver samples (Figure 10B). Enzymes associated with energy production were also noted, particularly the TCA cycle (NADPH, citrate synthase, and succinate exchange; *p* = 0.003, 0.013, and 0.014, respectively), ATP formation (2-deoxy-D-ribose 1-phosphate phosphorylase, deoxyribokinase, and deoxyribose-phosphate aldolase; all *p* = 0.003), and glycolysis (pyruvate carboxylase, enolase, and pyruvate kinase; *p* = 0.009, 0.013, and 0.013, respectively).

### 3.5. 16S rRNA Sequencing of Mouse Feces

**Figure 11:**
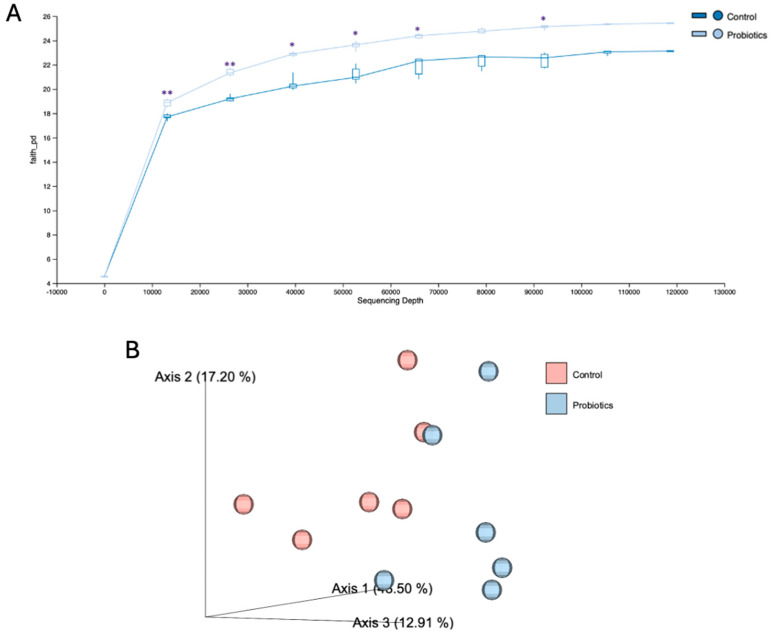
The effects of the probiotic cocktail on (**A**) α-diversity and (**B**) β-diversity of the gut microbiome using 16S rRNA sequencing. DNA was extracted from mouse fecal matter that was collected from C57BL/6J mice after the eight-week study. Analyses were performed in FASTQ format using QIIME2. Comparisons were between the control and the probiotic group. The graphs are presented as mean ± std. ** *p* <0.01, * *p* < 0.05.

Terminal fecal samples from C57BL/6J mice were collected to evaluate the probiotic effects on gut microbiota. In Figure 11A, the α-diversity quantifications demonstrated a significant change in the probiotic group, indicating improved evolutionary relationships within the microbiota. In Figure 11B, β-diversity of the control and probiotic groups is depicted in a principal coordinate analysis (PCoA) score plot. Clear separation between the groups can be observed with principal coordinate axis 1 representing the largest variation in microbial composition at 46.50% and coordinate axes 2 and 3 carrying 17.20% and 12.91%, respectively.

We analyzed the alterations of bacterial components in the mouse fecal samples (*n* = 6 per group) and reported the ten most significantly different taxa between groups. The relative frequency of gut microbes at the strain level in the control and probiotic groups is depicted in Figure S3. The 16S rRNA sequencing results showed a significant upregulation (*p* < 0.05) of *Lachnospiraceae bacterium 609-strain* and the genus *Muribaculaceae*, along with significant reductions (*p* < 0.05) in the *Odoribacter* species after probiotic supplementation. At the species level, the *Blautia* species and the genus *Muribaculaceae* were significantly increased in the probiotic group (Figure S4). At the genus level, significant increases were observed in *Lachnospiraceae* and *Muribaculaceae* after probiotic intake, while *Bacteriodaceae* was significantly decreased (Figure S5).

## 4. Discussion

In this study, we developed a novel approach to design a precision probiotic cocktail to increase gut microbial glucose consumption and reduce blood glucose levels in the host. First, we tested twelve bacterial strains after 24 h of in vitro incubation for their capabilities to consume glucose. In both MRS and GAM broths, *L. rhamnosus*, *L. reuteri*, and *L. salivarius* had the greatest measured glucose consumption. Then, a probiotic cocktail with these bacterial strains at approximately 5 × 10^8^ CFU/100 μL each was created and evaluated in C57BL/6J male mice on a high-fat diet over eight weeks, which is a well-established model for studying T2D and its associated metabolic changes, including hyperglycemia. The precision probiotic cocktail demonstrated a substantial reduction in the fasting blood glucose levels in comparison with the controls. In addition, the probiotic cocktail demonstrated the ability to reduce serum insulin, total cholesterol, VLDL/LDL cholesterol, and total cholesterol to HDL ratio. When paired together, significant improvements in glucose and insulin levels may represent early indicators of preventing insulin resistance. Furthermore, the mice receiving the probiotic cocktail had body fat percentages and consistent body weights that were significantly lower than the controls. Untargeted metabolomics analyses of serum and liver tissues uncovered significant group differences in energy substrates and producing pathways like gluconeogenesis, acylcarnitine synthesis, glycolysis, the mitochondrial electron transport chain, the TCA cycle, and the building blocks for ATP formation. Moreover, 16S rRNA sequencing revealed significant alterations to *Lachnospiraceae bacterium 609-strain*, *Odoribacter* species, and genus *Muribaculaceae* in the gut microbiome of the mice after 8 weeks of probiotic treatment. Our data support that the host molecular adaptations in the probiotic group to generate energy from noncarbohydrate sources could be due to a reduced glucose availability after enhanced gut bacterial consumption. In addition, our findings may demonstrate the potential benefits of reducing host glucose bioavailability through probiotics, which has the potential to mitigate hyperglycemia-related health conditions such as obesity and other metabolic disorders.

Poor glycemic control, including impaired fasting and/or postprandial blood glucose concentrations, is commonly associated with sedentary behavior and unhealthy dietary habits, including the consumption of foods high in added sugar and/or fat [5]. It has been reported that the average American currently consumes nearly two to three times the AHA recommendation for daily intake of added sugar [6]. This, coupled with other unhealthy dietary behaviors, is often referred to as the Western Diet. The Western Diet embodies calorically dense food that is sparse in nutrients [12,13]. Hyperglycemia is closely correlated with the development of obesity and metabolic syndrome, which may lead to severe conditions like T2D, CVD, and cancer [5,42]. In fact, nearly one-third of Americans have diabetes or prediabetes and struggle with poor glycemic control with incidence rates rising significantly [1,2]. Poor diet has also been shown to negatively impact the gastrointestinal tract and the gut microbiome. For instance, the Western Diet affects enterocyte tight junctions causing increased intestinal permeability resulting in elevated endotoxin levels in the bloodstream [20]. Poor dietary habits have also been associated with alterations to the gut microbiome composition, which plays vital roles in metabolism, immunity, inflammation, and the gut–brain axis [21,22]. Innovative strategies to protect the health of the gut microbiome and host from the harmful effects of high blood glucose levels are urgently needed.

In order to combat the negative health effects of poor glycemic control, researchers and companies have turned to probiotics as a potentially promising intervention. Although established probiotic brands claim similar benefits, the majority of them have not scientifically justified the selection of individual bacterial strains. Recent meta-analyses and reviews of studies have reported improvements in glucose regulation, cholesterol, and lipid profiles after probiotic supplementation, but the results have been inconsistent and varied [23,24,25]. This may be attributed to discrepancies in bacterial strains or dosages and highlights the need for in vitro experimentation to objectively select strains and dosages to achieve the desired benefit. Our objective was to identify the top glucose consuming bacterial strains in vitro and administer the novel precision probiotic to increase glucose consumption in the small intestine of the host. As a result, the potential bioavailability and absorption of glucose in the small intestine may be diminished, which may help mitigate the risk for related conditions.

The glucose-consuming strains used in our probiotic cocktail have reported benefits. *L. rhamnosus* is a strain commonly used as a probiotic for the prevention and treatment of gastrointestinal (GI) infections through improved gut motility and for alleviating allergic symptoms by immune response activation [43]. This bacterial strain is also thought to inhibit certain gut pathogens and protect the mucosa through biofilm production [44]. *L. reuteri* has been shown to enhance gut microbiome metabolic activity and improve blood glucose levels in rats with diabetes and metabolic syndrome [45,46]. A recent human study found that *L. reuteri* supplementation enhanced glucose-stimulated secretion of insulin and incretins glucagon-like peptides 1 and 2 (GLP-1 and GLP-2) [47]. *L. reuteri* has also been shown to produce antimicrobial metabolites, such as ethanol and organic acids, which help remodel the gut microbiome [48]. Furthermore, its other capacities, like increasing intestinal barrier strength, can reduce pathogen translocation and subsequently decrease tissue inflammation [48]. *L. salivarius* has also demonstrated protective metabolic effects and beneficial impacts on fasting blood glucose, glycemic control, and blood lipid profiles [46]. This type of bacteria is thought to activate glucose transporter 2 (GLUT2) expression, which is vital for the postprandial transport of glucose from the gut lumen into the blood stream [49]. Metabolites produced by *L. salivarius* have also been shown to possess antioxidant and antimicrobial effects that promote a healthy gut microbiome and host health [50]. The use of beneficial probiotic strains, shown to increase gut glucose consumption in this study, may be a feasible solution to reduce chronic poor glycemic control and associated complications.

To evaluate host metabolic adaptations resulting from the precision probiotic cocktail, serum samples were collected for metabolomic analyses. Several energy-producing pathways were impacted between the two groups. Many acylcarnitine derivatives (3-dehydrocarnitine, hexanoylcarnitine, 2-ethylacryloylcarnitine, and O-propanoylcarnitine), which are essential to long-chain fatty acid β-oxidation, were reduced after supplementation. Lower concentrations of circulating acylcarnitines are associated with healthy mitochondrial function [51,52,53]. These lower circulating concentrations may indicate increased cellular metabolic activity, particularly β-oxidation within the mitochondria. The breakdown of long-chain fatty acids for energy production can be attributed to reduced glucose availability, which was significantly lower in the probiotic group post treatment. An enrichment analysis of estimated enzyme activity also suggests an increase in oxygen transport within peroxisomes, organelles whose functions are related to β-oxidation and oxidative stress [54,55,56]. As a potential result of reduced glucose and pyruvic acid in the probiotic group, the metabolic pathway analysis indicated a significant shift in two pathways: (1) gluconeogenesis, a homeostatic mechanism involved in maintaining stable blood glucose concentrations through glucose biosynthesis from noncarbohydrate substrates; and (2) the glucose-alanine cycle [57]. These two pathway alterations may have impacted the final step of ATP production, the mitochondrial electron transport chain, which was also significantly different post treatment. While probiotic intake resulted in a greater demand for energy production, the control group had significantly elevated pathways related to fat storage pathways, such as de novo triacylglycerol biosynthesis, the shuttling of glycerol phosphate, and the exchange of the long chain fatty acid, arachidic acid. Interestingly, serum metabolites and enzymes related to vitamin C metabolism (L-threonic acid and L-ascorbate exchange, respectively) and vitamin B6 (pyridoxal transport and kinase) were also lower in the probiotic group and were identified as significant drivers of the PLS-DA score plot. The liver samples from the probiotic group demonstrated a significantly higher level of L-threonic acid compared to the control group, which opposes the serum findings and may indicate increased vitamin C metabolism in the host.

The liver is vital in many processes related to micronutrient and macronutrient metabolism [39]. The liver is also perpetually regulating blood glucose levels, especially when glucose intake does not meet energy demands [40,41]. As a potential mechanism for reduced serum glucose, the liver exhibited higher levels of the TCA cycle intermediates, oxoglutaric acid and malate. Metabolic pathway and enrichment analyses also revealed group differences in energy production. Metabolites related to carnitine synthesis for increased β-oxidation as well as the glucose-alanine cycle were significantly different between groups, which may be related to the diminished glucose availability after probiotic intake. Additionally, enzymatic activity associated with energy-producing pathways, like glycolysis, the TCA cycle, and ATP formation, was impacted. The liver also demonstrated greater group differences in amino acid metabolism than those in the serum. Probiotic supplementation resulted in higher levels of lysine and its intermediates with significantly lower levels of creatinine, which is noteworthy as it is the metabolic waste product of protein breakdown [58].

In order to assess probiotic interactions and the microbiome community, gut microbiota was extracted and 16S rRNA sequencing was performed between the two groups. After probiotic intake, the *Lachnospiraceae bacterium 609-strain* and the genus *Muribaculaceae* were significantly upregulated, while the *Odoribacter* species was downregulated. Many strains of *Lachnospiraceae* have been shown to reduce inflammation and are large producers of short-chain fatty acids (SCFAs) in the gut, which serve as a primary energy source for intestinal epithelial cells [59,60]. Similar increases in the genus *Muribaculaceae* have been associated with improved glucose regulation through activation of the insulin receptor/phosphatidylinositol-3-kinase/protein kinase B (IRS/PI3K/Akt) signaling pathway and increased SCFA production [61]. In addition, the probiotic cocktail resulted in significantly greater Faith’s phylogenetic diversity (α-diversity), which is calculated by the sum of the tree branch lengths and reflects the evolutionary relationships that connect all the observed taxa [62]. Moreover, a PCoA score plot depicting β-diversity demonstrated clear group differences in the microbial composition. While we did not see significantly different levels of our probiotic strains between groups, we did only report the ten most abundant taxa. It is possible that they were profoundly increased in the probiotic group but did not exceed ten other taxa. This comprehensive investigation of the probiotic-induced physiological adaptations offers potential mechanisms that may elucidate the observed host weight stability and improved blood glucose regulation. However, future clinical trials are warranted to better understand the effects of precision probiotics in the human body.

This study has limitations. First, only twelve male C57BL/6J mice were tested. Future research will aim to increase sample size and enhance generalizability of the findings by including both sexes and other species. In addition, this study included a short-term follow-up and did not consider energy intake or activity data as covariates. Future studies will consider a longitudinal design and mixed models with covariates. Despite the vast number of probiotic strains, this study only examined twelve strains that were hypothesized to be effective glucose consumers in the gut. We also recognize that this study was conducted in mice fed a high-fat diet, and further research using other diets, such as the Western Diet (high fat and high sugar), is necessary to further explore the reported benefits. The beneficial effects of the probiotic cocktail on gut homeostasis could also be investigated in future studies (e.g., along the gut–brain axis). Lastly, we acknowledge there are translational limits to mouse models and that clinical trials are required to confirm and better understand the benefits of this probiotic cocktail in real life.

## 5. Conclusions

In this study, we designed a protocol to develop a novel precision probiotic cocktail for blood glucose control and to reduce the risk of hyperglycemia-associated diseases. We identified three bacterial strains, *L. rhamnosus*, *L. reuteri*, and *L. salivarius*, which were the most efficient consumers of glucose. We discovered that the probiotic cocktail blunted elevations in fasting blood glucose and body weight in C57BL/6J mice fed a high-fat diet for eight weeks compared to the control group. Moreover, the probiotic group demonstrated significant improvements in circulating fasting insulin, total cholesterol, and VLDL/LDL concentrations as well as body fat percentage. Metabolomic analyses indicated host metabolic adaptations for energy-producing pathways like gluconeogenesis, glycolysis, the TCA cycle, and oxidative phosphorylation. In addition, the 16S rRNA sequencing results showed significant increases in Faith’s phylogenetic/α diversity, the *Lachnospiraceae bacterium 609-strain*, and the genus *Muribaculaceae* after probiotic intake. We present a proof-of-concept study demonstrating that our precision probiotic cocktail suggests potential in increasing gut glucose consumption and thereby reducing glucose bioavailability to the host. It may be surmised that precision probiotics may potentially reduce the risk for adverse health conditions associated with poor glycemic control, and future clinical trials are needed to confirm our results.

## Data Availability

The data presented in this study are available on request from the corresponding author due to a filed patent for this work.

## Supplementary Materials

The following supporting information can be downloaded at: https://www.mdpi.com/article/10.3390/metabo15100642/s1, Figure S1: Serum metabolite volcano plot analysis comparing the control and probiotic groups of C57BL/6J male mice on a high-fat diet for 8 weeks; Figure S2: Liver metabolite volcano plot analysis comparing the control and probiotic groups of C57BL/6J male mice on a high-fat diet for 8 weeks; Figure S3: The effects of the probiotic cocktail on the gut microbiome using 16S rRNA sequencing at the strain level; Figure S4: The effects of the probiotic cocktail on the gut microbiome using 16S rRNA sequencing at the species level; Figure S5: The effects of the probiotic cocktail on the gut microbiome using 16S rRNA sequencing at the genus level; Table S1: Serum significant metabolites and fold change analysis comparing the control and probiotic groups of C57BL/6J male mice on a high-fat diet for eight weeks; Table S2: Liver significant metabolites and fold change analysis comparing the control and probiotic groups of C57BL/6J male mice on a high-fat diet for eight weeks.
