# Supplementary material for: Precision Probiotics Regulate Blood Glucose, Cholesterol, Body Fat Percentage, and Weight Under Eight-Week High-Fat Diet
Source: Metabolites. 2025 Sep 25;15(10):642. doi: 10.3390/metabo15100642 (PMC12566540; doi:10.3390/metabo15100642)
Supplement: Supplementary file 1 [file metabolites-15-00642-s001.zip › metabolites-3848206-supplementary.pdf]

Supplemental Information

Precision Probiotics Regulate Blood Glucose, Cholesterol, Body Fat Percentage, and Weight under Eight-Week High-Fat Diet

Jinhua Chi[1#], Jeffrey S. Patterson[1#], Lingjun Li[1], Nicole Lalime[2], Daniella Hawley[3], Kyle Joohyung Kim[4], Li Liu[1], Julia Yue Cui[4], Dorothy D. Sears[1], Paniz Jasbi[5,6], Haiwei Gu[1,6*]

[1]College of Health Solutions, Arizona State University, Phoenix, AZ 85004, USA

[2]School of Biological and Health Systems Engineering, Arizona State University, Tempe, AZ 85287, USA

[3]School of Life Sciences, Arizona State University, Tempe, AZ 85287, USA

[4]Department of Environmental & Occupational Health Sciences, University of Washington, Seattle, WA 98195, USA

[5]Systems Precision Engineering and Advanced Research (SPEAR), Theriome Inc., Phoenix AZ, 85004, USA

[6]MetaBiotics LLC, Scottsdale, AZ 85259, USA

[#]Jinhua Chi and Jeffrey S. Patterson are co-first authors

*Corresponding Author:

Haiwei Gu, PhD

College of Health Solutions

Arizona State University

850 N 5th Street

Phoenix, AZ 85004

Email: haiweigu@asu.edu

Tel: 480-301-6016

Figure S1: Serum metabolite volcano plot analysis comparing the control and probiotic groups of C57BL/6J male mice on a high-fat diet for 8 weeks

Figure S2: Liver metabolite volcano plot analysis comparing the control and probiotic groups of C57BL/6J male mice on a high-fat diet for 8 weeks

Figure S3: The effects of the probiotic cocktail on the gut microbiome using 16S rRNA sequencing at the strain level

Figure S4: The effects of the probiotic cocktail on the gut microbiome using 16S rRNA sequencing at the species level

Figure S5: The effects of the probiotic cocktail on the gut microbiome using 16S rRNA sequencing at the genus level

Table S1: Serum significant metabolites and fold change analysis comparing the control and probiotic groups of C57BL/6J male mice on a high-fat diet for eight weeks

Table S2: Liver significant metabolites and fold change analysis comparing the control and probiotic groups of C57BL/6J male mice on a high-fat diet for eight weeks

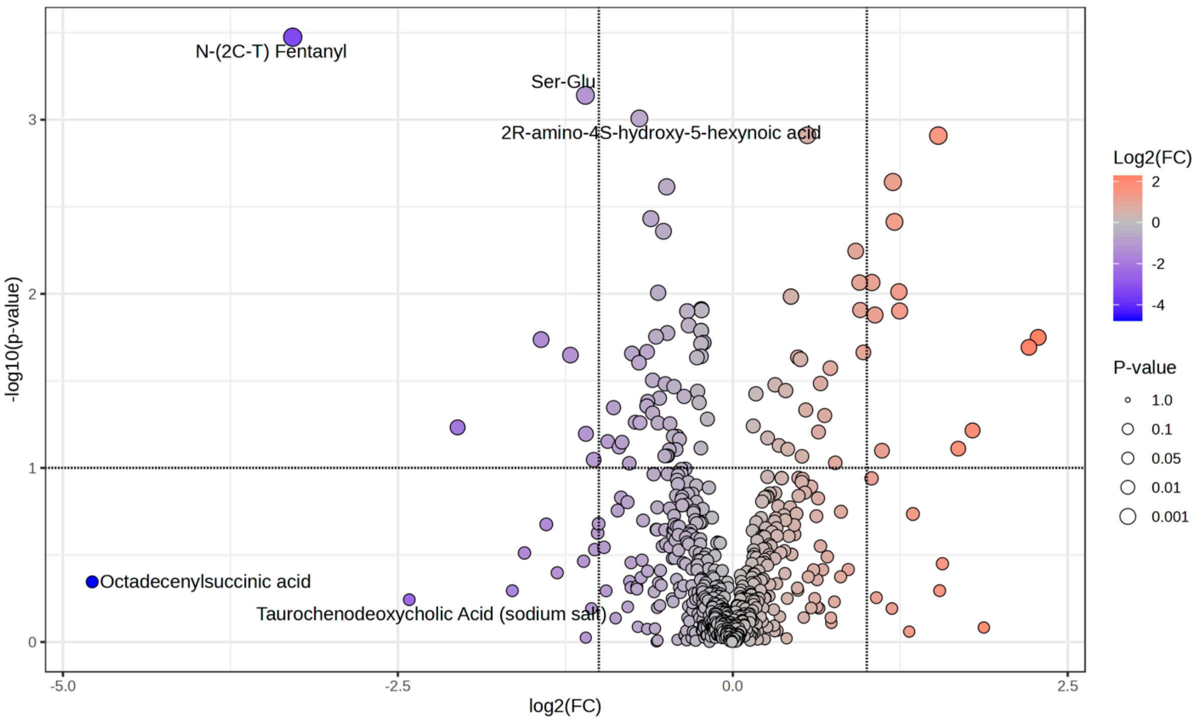

Figure S1: Serum metabolite volcano plot analysis comparing the control and probiotic groups of C57BL/6J male mice on a high-fat diet for 8 weeks. Fold changes are plotted as Probiotic/Control samples.

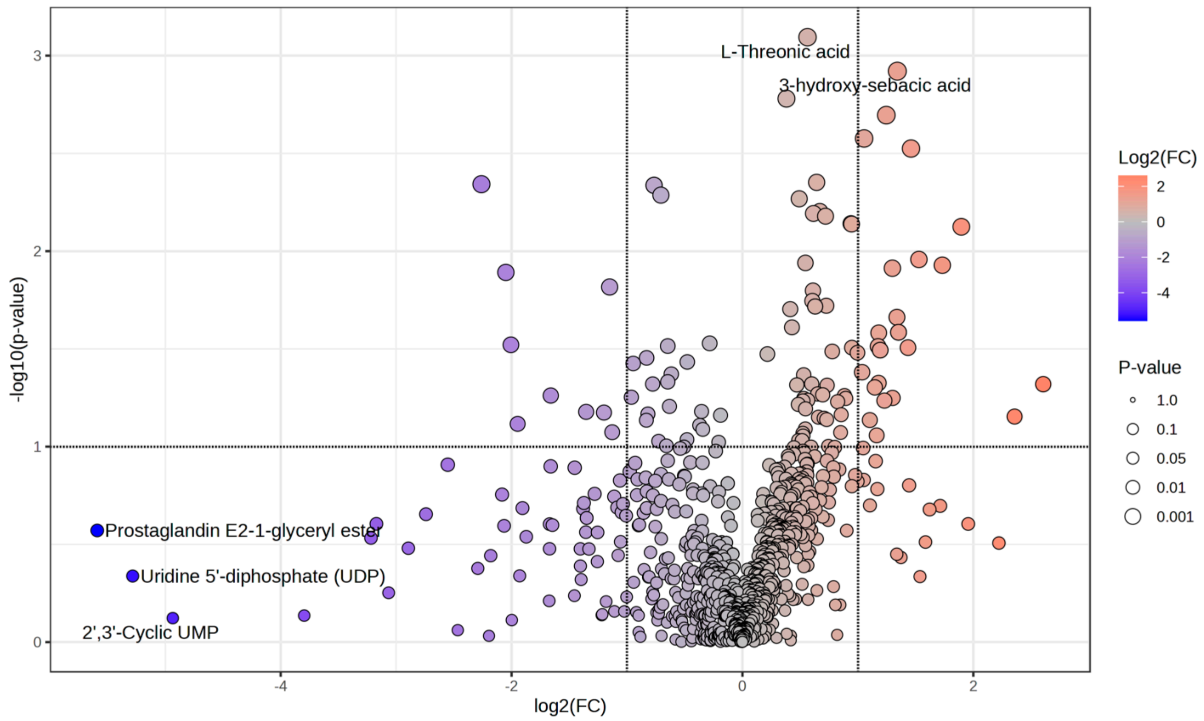

Figure S2: Liver metabolite volcano plot analysis comparing the control and probiotic groups of C57BL/6J male mice on a high-fat diet for 8 weeks. Fold changes are plotted as Probiotic/Control samples.

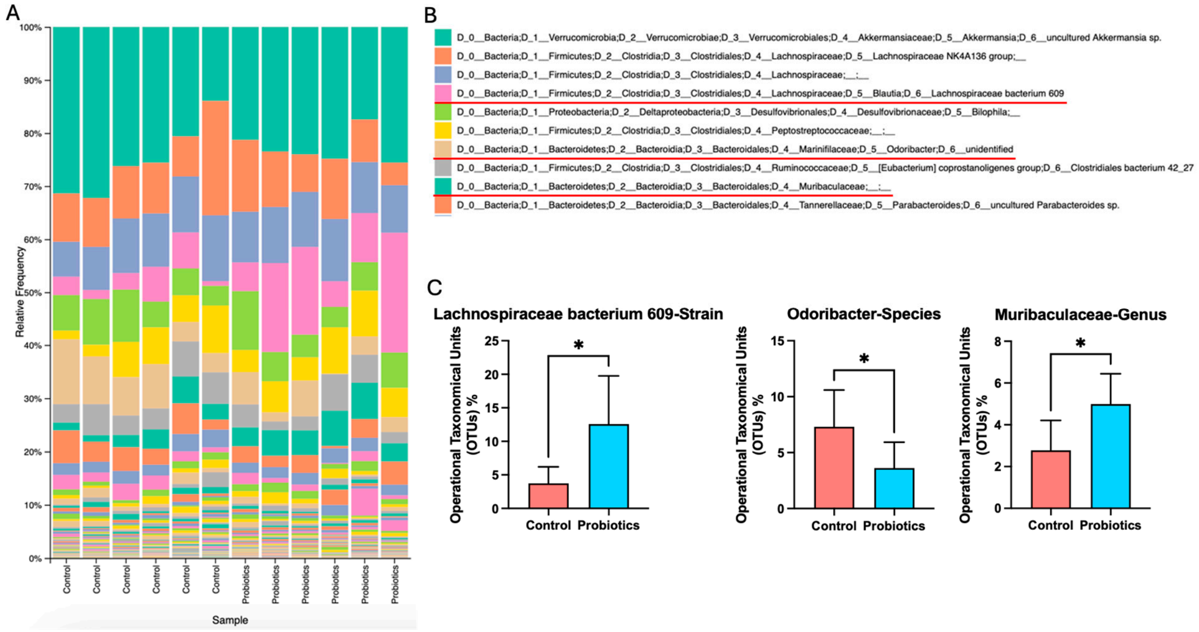

Figure S3. The effects of the probiotic cocktail on (A) the relative frequency of gut microbes at the strain level in the control and probiotic groups, (B) the ten most abundant taxa identified by QIIME2 (red line indicates analyzed taxa), and (C) the operational taxonomical units of *Lachnospiraceae* bacterium 609-strain, *Odoribacter* species, and genus *Muribaculaceae.* DNA was extracted from mouse fecal matter that was collected from C57BL/6J mice after the eight-week study. Analyses were performed in FASTQ format using QIIME2. Comparisons were between the control and the probiotic group. The graphs are presented as mean ± std. * $P < 0.05$.

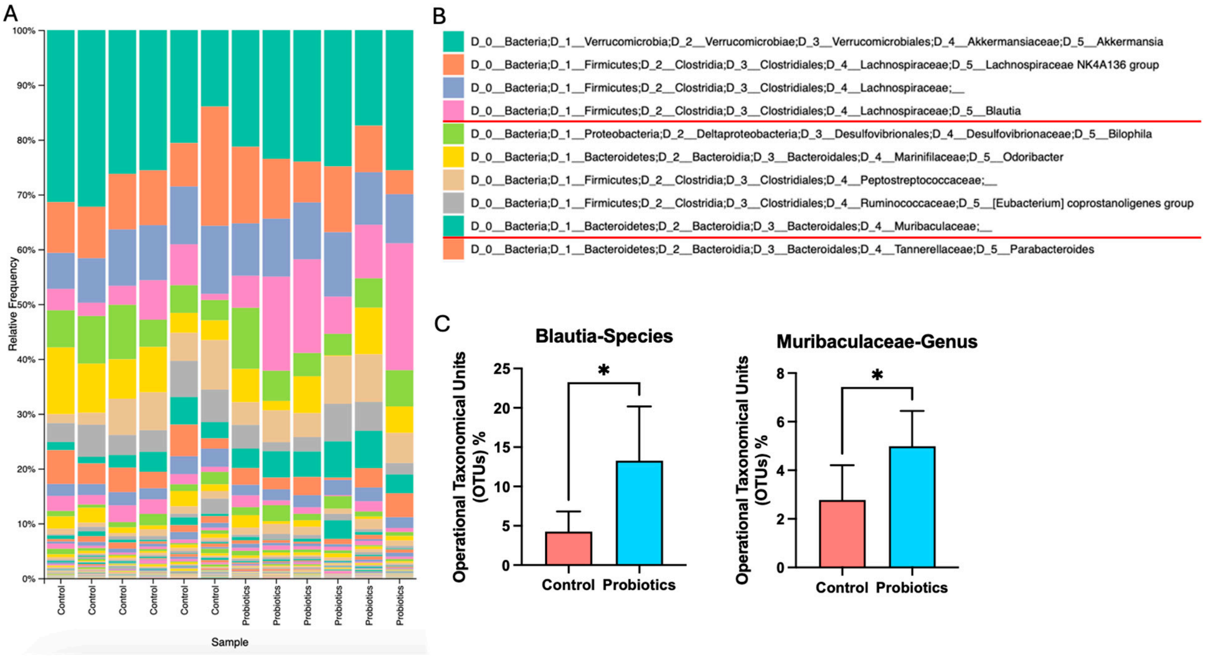

Figure S4. The effects of the probiotic cocktail on the gut microbiome using 16S rRNA sequencing at the species level. (A) The relative frequency of gut microbes at the species level from the control and probiotic groups, (B) ten most abundant taxa identified by QIIME2 (red line indicates analyzed taxa), and (C) operational taxonomical units of *Blautia* species and *Muribaculaceae* genus in control vs. probiotic groups, * $P < 0.05$

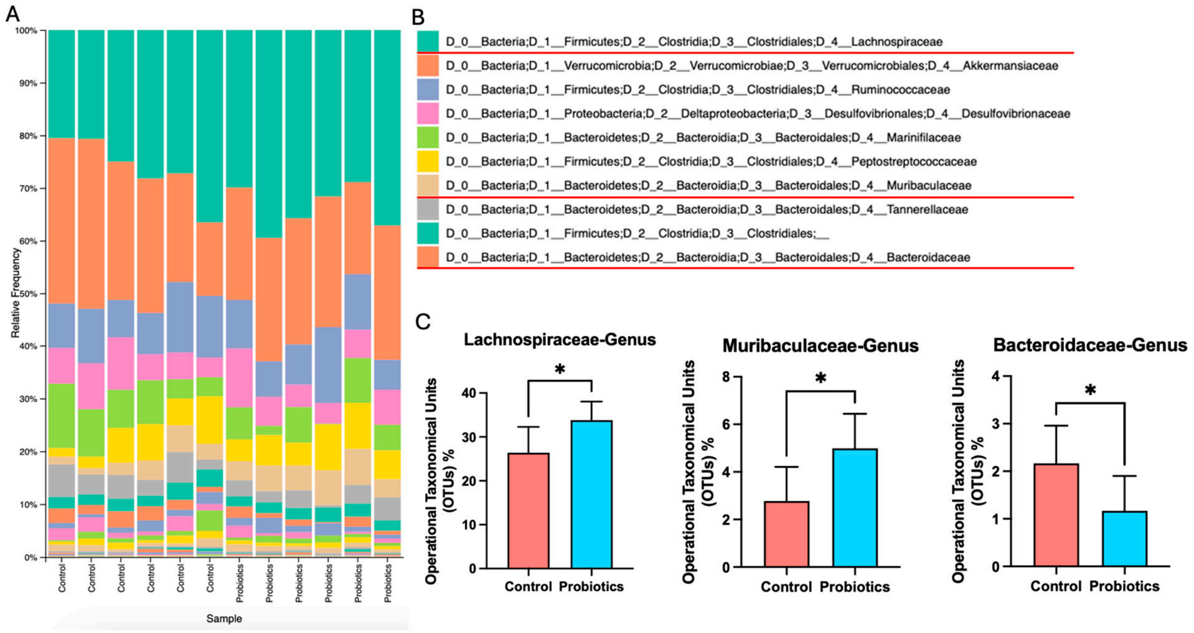

Figure S5. The effects of the probiotic cocktail on the gut microbiome using 16S rRNA sequencing at the genus level. (A) The relative frequency of gut microbes at the genus level from the control and probiotic groups, (B) ten most abundant taxa identified by QIIME2 (red line indicates analyzed taxa), and (C) operational taxonomical units of *Lachnospiraceae*, *Muribaculaceae*, and *Bacteriodaceae* in control vs. probiotic groups, * $P < 0.05$

Table S1: Serum significant metabolites and fold change analysis comparing the control and probiotic groups of C57BL/6J male mice on a high-fat diet for eight weeks.

Metabolite	Fold Change	*P*-value
N-(2C-T) Fentanyl	0.1026	0.0003356
Ser-Glu	0.46647	0.00072395
2R-amino-4S-hydroxy-5-hexynoic acid	0.61669	0.00098255
N-Isovalerylglycine	1.4706	0.0012283
Botryosphaerilactone A	2.896	0.0012332
Methacrylic acid, ester withmethoxytriethyleneglycol	2.2897	0.0022784
Bufexamac	0.71029	0.0024294
5-hydroxy-octanoic acid	0.65442	0.0037002
Diethylene glycol diglycidyl ether	2.3084	0.0038618
Uric acid	0.69856	0.0043729
1_2-Dihydrosantonin	1.8888	0.0056818
4-amino-2-hydroxyamino-6-nitrotoluene	2.0528	0.0086131
Aspergone N	1.9265	0.0086164
Dibutyl thiourea	2.3619	0.0097359
L-Glutamic acid	0.67957	0.0098622
3-Dehydrocarnitine	1.3509	0.010375
4-(2-hydroxyethyl)-5-methyloxazole	1.3509	0.010375
3S,4-dihydroxy-butyric acid	0.84951	0.012252
(E)-2-(hydroxymethyl)-3-(4-hydroxypent-1-enyl)phenol	1.9337	0.012393
D-(+)-Glucose	0.85049	0.012424
But-3-en-1-amine	2.3709	0.012546
2-Oxo-delta3-4_5_5-trimethylcyclopentenylacetate	0.7899	0.0126
Ecgoninemethylester	0.7899	0.0126
9,10-dihydroxy-2-decenoic acid	2.0873	0.013231
Hexanoylcarnitine	0.79607	0.015188
4-Hydroxy-2-butynal	0.84876	0.016314
Ethyl maleate	0.84876	0.016314
4-hydroxy capric acid	0.71262	0.016797
Trehalamine	0.67234	0.017623
4-Phenolsulfonic acid	4.8557	0.017792
Triethanolamine	0.37084	0.018319
(S)-4-hydroxy-2-oxopentanoic acid	0.85994	0.019076
Diacetyl	0.85994	0.019076
2-Hydroxyadipic acid	0.84871	0.019324
Phenol	4.628	0.020299
D-(+)-Malic acid	0.64179	0.021536

Bufotenine	1.9651	0.021701
D-Ribose-1-phosphate	0.59335	0.022
L-Threonicacid	0.43165	0.022491
2-Hydroxy-2_4-pentadienoate	0.84824	0.022864
Aspilactonol G	1.4009	0.023165
Hydracrylic acid, acrylate	0.83061	0.023223
Aldicarb-sulfone	1.4188	0.023776
N-Methylglucamine	0.6155	0.024878
2-ethylacryloylcarnitine	1.6564	0.026727
1,3,5-Tris(2-hydroxyethyl)cyanuric acid	0.66051	0.031369
Glycerol monobutyrate	1.5743	0.03276
O-Propanoylcarnitine	0.70516	0.032905
Capryloylglycine	1.2445	0.03331
(S)-9-Hydroxy-10-undecenoic acid	0.73751	0.034136
4-oxo-2E-nonenoic acid	1.3147	0.035953
2-Oxovalericacid	0.83352	0.036337
Betaine	1.127	0.037535
5-L-Glutamyl-taurine	0.77748	0.038862
N-hexanoyl-homoserine lactone	0.68408	0.039767
N-Acetylglucosaminitol	0.64397	0.041505
D-(+)-Mannose	0.84026	0.042183
(2R)-2,3-Dihydroxypropanoic acid	0.64127	0.044144
Pyruvic acid	0.64127	0.044144
Myristyl sulfate	0.53951	0.045086
2-[4-(tert-butyl)-2,3-dihydro-1,3-thiazol-2-yliden]-3-oxobutanenitrile	1.4587	0.046479
2-Furoylglycine;Pyromucuricacid	0.66046	0.048398

Note. Fold Change analysis was calculated as Probiotic/Control samples.

Table S2: Liver significant metabolites and fold change analysis comparing the control and probiotic groups of C57BL/6J male mice on a high-fat diet for eight weeks.

Metabolite	Fold Change	*P*-value
L-Threonic acid	1.4779	0.00080474
3-hydroxy-sebacic acid	2.534	0.0012006
Aminoadipic acid	1.3024	0.0016594
9,10-dihydroxy-2-decenoic acid	2.3717	0.0020154
2-Oxoglutaric acid	2.0762	0.0026531
Zolpidem	2.7514	0.002987
N-Acetyl-L-glutamate5-semialdehyde	1.5611	0.0044483
Gamma-Glutamylcysteine	0.20859	0.0045458
2-[[(Butylamino)carbonyl]oxy]ethyl acrylate	0.58814	0.0046059
O6-Cyclohexylmethylguanine	0.58814	0.0046059
N1-(1,3,5-Trimethyl-1H-pyrazol-4-yl)-2-cyano-3-(dimethylamino)acrylamide	0.61285	0.0051713
3-methyl-adipic acid	1.4062	0.0053974
delta-Valerolactone	1.5914	0.0062678
Garcinia acid	1.5316	0.0064177
2,5-Diaminopentanoic acid	1.6468	0.0066261
2-amino-3-oxo-hexanedioic acid	1.9185	0.0072315
2,Ä≤-deamino-2,Ä≤-hydroxy-6,Ä≤-dehydroparomamine	1.9259	0.0072717
L-Homocysteicacid	3.7221	0.007505
Isocarbamid	2.8851	0.01103
2R-amino-4S-hydroxy-5-hexynoic acid	1.4595	0.011489
N3-Oxalyl-L-2_3-diaminopropanoate	3.3203	0.011806
N-butyryl-L-Homocysteine thiolactone	2.46	0.012229
DL-3-Aminoisobutyric acid	0.24155	0.012846
3-Amino-4,7-dihydroxy-8-chlorocoumarin	0.45047	0.015258
Methylmalonic acid	1.5272	0.015922
L-Lysine	1.5217	0.017986
4,6-dihydroxy-2,3-dihydro-1H-isoindol-1-one	1.6543	0.019023
2-Propynylamine, 1,1-dimethyl-	1.5474	0.019186
Piperazine, 1-methyl-	1.5474	0.019186
N6-Acetyl-L-lysine	1.3324	0.019804
3-Isopropyl-6-methyl-2,5-piperazinedione	2.5282	0.021789
carglumic acid	1.3461	0.024535
N-(2-Methoxyethyl)-3-{5-[(2S)-1-methyl-2-pyrrolidinyl]-1,2,4-oxadiazol-3-yl}-2-pyridinamine	2.5525	0.02601
Streptidine	2.5525	0.02601
7-(2,4-Dinitro-phenoxy)-4-methyl-chromen-2-one	2.2664	0.026178

19-(2-acetamido-2-deoxy-Œ±-D-glucopyranosyloxy)isopimara-7,15-dien-3Œ≤-ol	0.8214	0.029632
11-Aminoundecanoic acid	0.249	0.030145
2-benzoyl-3-(2-pyridylamino)acrylonitrile	0.63858	0.030579
Salicylamide	2.2577	0.030728
2-amino-muconic acid	2.7019	0.031157
N-(4-Amino-1-carboxybutyl)glutamic acid	1.9292	0.031167
Cispentacin	2.2866	0.032091
R-(+)-Etiracetam	2.2866	0.032091
3-amino-4,5,6-trihydroxy-2-methoxy-5-methyl-2-cyclohexen-1-one	1.7147	0.032589
Malonic acid	1.995	0.033094
1-Myristoyl-2-linoleoyl-sn-glycero-3-phosphocholine	1.1616	0.033604
Creatinine	0.56247	0.035162
Phosphoric acid, mono(2-ethylhexyl) ester	0.71802	0.036885
9E,11-Dodecadienal	0.51896	0.037548
3-Ureidoisobutyrate	2.0515	0.041621
Ethyl acetoacetate	0.65226	0.042559
Terminalin	1.4444	0.042984
Ethyl Butylacetylaminopropionate	0.63856	0.046617
1-Aminocyclohexanecarboxylic Acid	2.2641	0.047275
1,4-Dioxacyclotridecane-5,13-dione	1.5156	0.047777
Seliciclib	0.58333	0.047833
Oxalamido-L-leucine methyl ester	6.0858	0.047922
N-decanoyl-homoserine lactone	1.386	0.04833
Itatartaric acid	1.6625	0.048561

Note. Fold Change analysis was calculated as Probiotic/Control samples.
